# High-speed automatic characterization of rare events in flow cytometric data

**DOI:** 10.1371/journal.pone.0228651

**Published:** 2020-02-11

**Authors:** Yuan Qi, Youhan Fang, David R. Sinclair, Shangqin Guo, Meritxell Alberich-Jorda, Jun Lu, Daniel G. Tenen, Michael G. Kharas, Saumyadipta Pyne

**Affiliations:** 1 Department of Computer Science, Purdue University, West Lafayette, IN, United States of America; 2 Department of Statistics, Purdue University, West Lafayette, IN, United States of America; 3 Population Health Sciences Institute, Newcastle University, Newcastle upon Tyne, United Kingdom; 4 Public Health Dynamics Laboratory, Graduate School of Public Health, University of Pittsburgh, Pittsburgh, PA, United States of America; 5 Department of Health Policy and Management, Graduate School of Public Health, University of Pittsburgh, Pittsburgh, PA, United States of America; 6 Department of Cell Biology, Yale University School of Medicine, New Haven, CT, United States of America; 7 Institute of Molecular Genetics of the ASCR, Prague, Czech Republic; 8 Department of Genetics, Yale University School of Medicine, New Haven, CT, United States of America; 9 Yale Stem Cell Center, Yale University School of Medicine, New Haven, CT, United States of America; 10 Center for Life Sciences, Harvard Medical School, Boston, MA, United States of America; 11 Harvard Stem Cell Institute, Harvard Medical School, Boston, MA, United States of America; 12 Cancer Science Institute, National University of Singapore, Singapore, Singapore; 13 Molecular Pharmacology Program, Memorial Sloan Kettering Cancer Center, New York, NY, United States of America; 14 Department of Biostatistics, Graduate School of Public Health, University of Pittsburgh, Pittsburgh, PA, United States of America; Stanford University, UNITED STATES

## Abstract

A new computational framework for FLow cytometric Analysis of Rare Events (FLARE) has been developed specifically for fast and automatic identification of rare cell populations in very large samples generated by platforms like multi-parametric flow cytometry. Using a hierarchical Bayesian model and information-sharing via parallel computation, FLARE rapidly explores the high-dimensional marker-space to detect highly rare populations that are consistent across multiple samples. Further it can focus within specified regions of interest in marker-space to detect subpopulations with desired precision.

## Introduction

Studies focusing on rare cell populations are becoming increasingly common owing to technological advances such as high-speed, multi-parametric flow cytometry, and emerging biomedical applications like stem cell therapy, and single cell analysis. Researchers in fields such as hematology, cancer, immunology, pathology, stem cell biology, and regenerative medicine, have focused on many interesting, yet relatively rare, populations of cells in blood and other tissues and systems that have important biomedical functions and characteristics, e.g., long-term hematopoietic stem cells.

Methods for accurate detection or automated isolation of rare therapy-resistant cells in tumors with stem cell like properties, tumor cells circulating in blood, or regulatory T cells, can have profound influence on basic and clinical research. Platforms like multi-color flow cytometry, in conjunction with the development of diverse panels of markers and antibodies, have been used to establish signatures for various rare cellular species and lineages in terms of the expressed surface and intracellular marker proteins [1]. Advances in mass cytometry have promised the ability to determine 50-100 features per cell [2, 3]. To address such increasingly multi-parametric and multiplexed immunoprofiling of each cell, studies have demonstrated the critical need for systematic and automated multivariate analysis and visualization suitable for high-dimensional data [4–6]. As the number of potential combinations of markers continues to grow exponentially (with the number of markers), a thorough search for rare events in high-dimensional marker-space clearly gets difficult with the more subjective and painstaking approach of traditional manual gating [7].

Analytically, a population of cells having similar, characteristic expression of *k* (> 1) markers can be measured as events with similar fluorescence intensities, i.e., as a cluster of points located closely in *k*-dimensional marker-space [4]. However traditional clustering approaches may not be adequate for identification of rare cell populations for several technical reasons. The new data are not only high-dimensional (i.e., involving multi-parametric or multiplexed panels) but simultaneously are also high-resolution (single cell level) and considerably high-throughput (hundreds of thousands of cells per sample) by design. Typically, therefore, if a population of interest is rare and consists of, say, fewer than 1% or 0.1% of the total number of cells in a given sample, then for reliable detection of such a population, it is common to use a sample size (*N*) in the order of 10^5^ − 10^6^ cells, each measured as a *k*-dimensional point. Thus a large cytometric sample can present a “searching for a needle in a haystack” scenario for the identification of any rare population therein, resulting either in inefficient coverage of the *k*-dimensional marker-space (the volume of which increasing exponentially with *k*), or detection of a number of spurious small populations (often outliers of larger, noisy populations). In general, clustering methods like k-means or hierarchical clustering use some measure of distance between every pair of points to determine their closeness for clustering assignment. While effective for clustering a few thousands genes or features in omic data, clearly such quadratic-time *O*(*N*^2^) approaches would be computationally inadequate for searching complex cytometric datasets with much larger *N*.

Another practical challenge stems from biological and/or technological sources of inter-sample variation including single cell level heterogeneity, individual subjects, different time-points and conditions, and platform noise—all of which make consistent identification of particularly the rarer populations difficult. Moreover, as cells undergo state transitions, for instance during differentiation, the corresponding changes in marker-expressions result in hierarchies of inter-connected clusters. Such clusters may contain complex high-dimensional structures such as heavy tails or skewness, that present unique data modeling challenges for computational analysis [5, 8]. Therefore, we developed FLARE as a new computational framework that can simultaneously meet the somewhat conflicting requirements of (a) high speed, (b) high precision, and (c) robust data modeling.

## Model

In this section, we describe the our new hierarchical Bayesian model, FLARE, for FLow cytometric Analysis of Rare Events, to identify cell populations from multiple samples and detecting rare cell populations. Given the increasing high-dimensionality of cytometric data, there is a critical need to assist the manual gating procedure using unsupervised computational approaches to explore the marker-space, especially to identify specific cell populations that may appear at unknown locations under certain conditions such as drug-resistant cells or a rare signature of disease prognosis.

To this end, we designed a hierarchical Bayesian model that can share information across multiple samples to substantiate the occurrence of any genuine rare cluster of events. First, we model the cell populations in each sample by a mixture of probability distributions, say, multivariate Gaussian components, so that we can assign a probability score to associate each cell with a population, thus reflecting the underlying structures of individual samples. Second, we let the Gaussian components—corresponding to cell populations in different samples—be similar to each other via common prototype populations up to certain small variations, so that we can capture the minor differences between individual samples. Third, we allow some Gaussian components to appear only in certain—but not necessarily all—samples, and report these populations, even if they are rare events.

Let us denote the cytometric data by **X** and the cell memberships by $H=h_{nk}^{\left(m\right)}$ where $h_{nk}^{\left(m\right)}$ denotes the membership of the *n*-th cell in the *m*-th sample to that sample’s *k*-th Gaussian component with mean $\mu_{k}^{\left(m\right)}$ and precision $\lambda_{k}^{\left(m\right)}$. Then the data likelihood is

$$\begin{array}{c} P\left(X|\Theta ,H\right)=\prod_{m=1}^{M}\prod_{n=1}^{N_{m}}\prod_{k=1}^{K}N{\left(x_{n}^{\left(m\right)}|\mu_{k}^{\left(m\right)},{\left(\Lambda_{k}^{\left(m\right)}\right)}^{-1}\right)}^{h_{nk}^{\left(m\right)}} \end{array}$$ (1)

where *M* is the number of samples, *N*_*m*_ the number of cells in sample *m*, *K* the maximal number of cell populations for each sample, and $\Theta ={\left\{\mu_{k}^{\left(m\right)},\Lambda_{k}^{\left(m\right)}\right\}}_{m,k}$. Each data point $x_{n}^{\left(m\right)}$ has dimension *D*.

The latent membership indicators **H** has a factorized discrete prior distribution:

$$\begin{array}{c} P\left(H|\pi \right)=\prod_{m=1}^{M}\prod_{n=1}^{N_{m}}\prod_{k=1}^{K}{\left(\pi_{k}^{\left(m\right)}\right)}^{h_{nk}^{\left(m\right)}} \end{array}$$ (2)

where $\pi_{k}^{\left(m\right)}$ is the probability of the *k*-th population appearing in sample *m* and $\sum_{k}\pi_{k}^{\left(m\right)}=1$. If $\pi_{k}^{\left(m\right)}=0$, then the *k*-th population does not exist in the *m*-th sample. To model the uncertainty in $\pi^{\left(m\right)}=\left[\pi_{k}^{\left(m\right)},\ldots ,\pi_{k}^{\left(m\right)}\right]$, we use a symmetric Dirichlet prior distribution:

$$\begin{array}{c} P\left(\pi \right)=\prod_{m=1}^{M}C\left(\alpha_{0}\right)\prod_{k=1}^{K}{\left(\pi_{k}^{\left(m\right)}\right)}^{\alpha_{0}-1} \end{array}$$ (3)

where *α*_0_ is a hyperparameter and $C\left(\alpha_{0}\right)=\frac{\Gamma (M\alpha_{0})}{\Gamma {\left(\alpha_{0}\right)}^{M}}$.

To share information between clusters of different samples, we let the mean parameter, $\mu_{k}^{\left(m\right)}$, of each cluster in a sample follow a Gaussian prior distribution common to all samples:

$$\begin{array}{c} P\left(\mu_{k}^{\left(m\right)}|\eta_{k}\right)=N\left(\mu_{k}^{\left(m\right)}|\eta_{k},{\left(\beta_{0}I\right)}^{-1}\right) \end{array}$$ (4)

where ***η***_*k*_ is the mean parameter of the *k*-th prototype cluster—which is estimated from data as $\mu_{k}^{\left(m\right)}$—and *β*_0_ is a hyperparameter. Similar, the covariance matrix, $\Lambda_{k}^{\left(m\right)}$, of each cluster in a sample follows a Wishart prior distribution common to all samples:

$$\begin{array}{c} P\left(\Lambda_{k}^{\left(m\right)}|\Omega_{k}\right)=W\left(\Lambda_{k}^{\left(m\right)}|\Omega_{k},\sigma_{0}\right) \end{array}$$ (5)

where **Ω**_*k*_ is a symmetric, positive definite matrix—estimated from data just as $\Lambda_{k}^{\left(m\right)}$—and *σ*_0_ is the degree of freedom.

Since we need to estimate the parameters of the prototype clusters from data as well, we assign a Gaussian hyper-prior distribution over the mean of each prototype cluster, ***η***_***k***_:

$$\begin{array}{c} P\left(\eta_{k}\right)=N{\left(\eta_{k}|0,I\right)}^{-1}) \end{array}$$ (6)

Also, we assign an Inverse-Wishart hyper-prior distribution over the shape of each each prototype cluster, **Ω**:

$$\begin{array}{c} P\left(\Omega_{k}\right)=W^{-1}\left(\Omega_{k}|\Phi_{0},\nu_{0}\right) \end{array}$$ (7)

where **Φ**_0_ and *ν*_0_ are hyperparameters. In our experiments, we set **Φ**_0_ = **I** and *ν*_0_ = 6*D* to obtain a diffuse prior over **Ω**_*k*_.

Combining the data likelihood, the priors and the hyper-priors, we obtain the following joint distribution for our model:

$$\begin{array}{c} P(X,\mu ,\Lambda ,\eta ,\Omega ,h,\pi )\\ =P(X|\mu ,\Lambda ,h)P(h|\pi )P(\mu |\eta )P(\Lambda |\Omega )P(\eta )P(\Omega )P(\pi )\\ =(\prod_{m=1}^{M}\prod_{n=1}^{N_{m}}\prod_{k=1}^{K}N{\left(x_{n}^{\left(m\right)}|\mu_{k}^{\left(m\right)},{\left(\Lambda_{k}^{\left(m\right)}\right)}^{-1}\right)}^{h_{nk}^{\left(m\right)}}\prod_{m=1}^{M}\prod_{n=1}^{N_{m}}\prod_{k=1}^{K}{\left(\pi_{k}^{\left(m\right)}\right)}^{h_{nk}^{\left(m\right)}})\\ \left(\prod_{k=1}^{K}N{\left(\eta_{k}|0,I\right)}^{-1})W^{-1}\left(\Omega_{k}|\Phi_{0},\nu_{0}\right)\right) \end{array}$$ (8)

**Fig 1:**
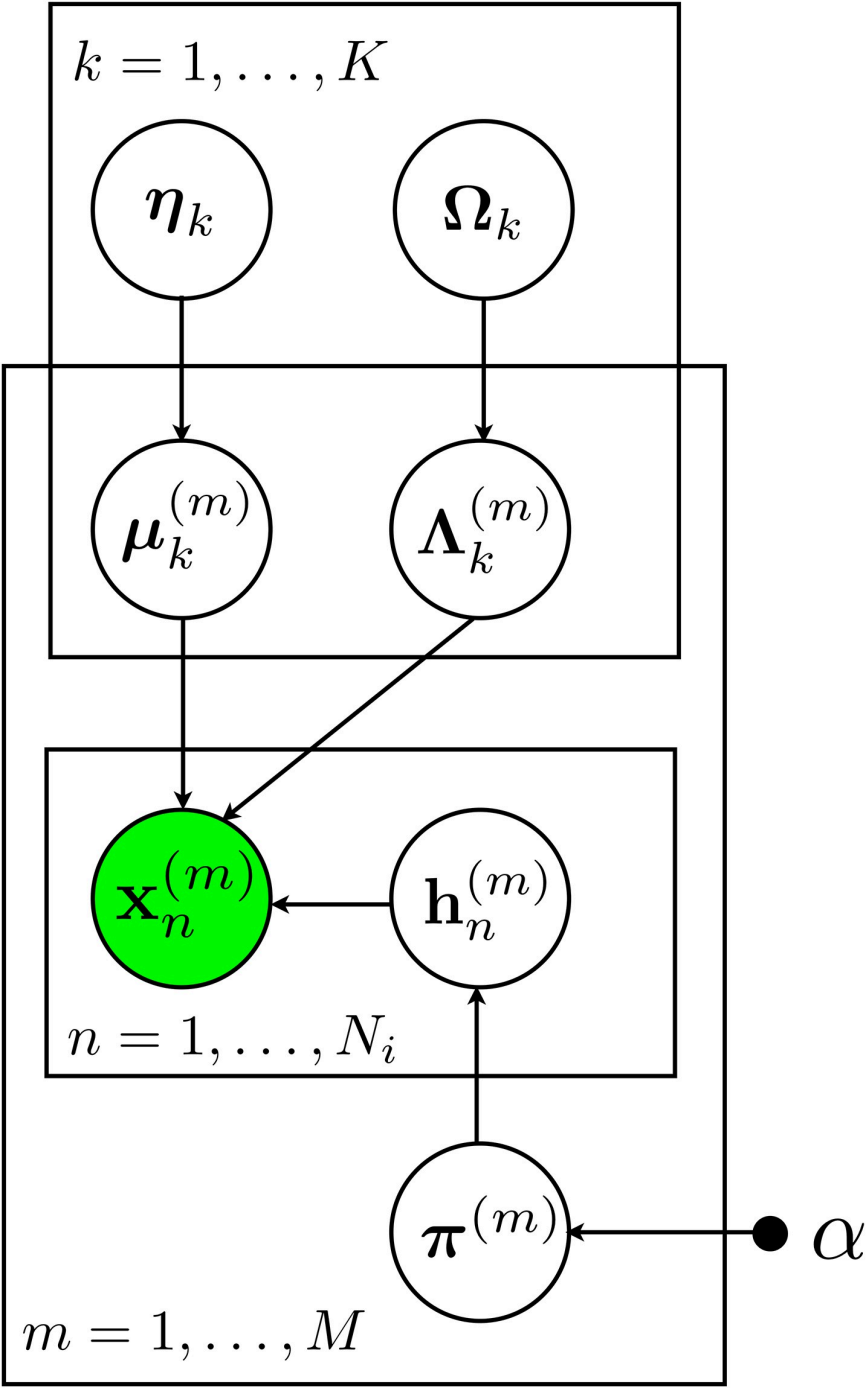
The probabilistic graphical model representation of FLARE. *x* is the cell, *μ* and Λ are the mean and covariance of the parent clusters, *η* and Ω are the mean and covariance of cluster *k*, *π* is the membership distribution, *h* is the membership indicator and *α* is the hyperparameter for *π*. The superscript *m* indicates the *m*^th^ sample.

The joint distribution is depicted in Fig 1.

With the priors specified over the cluster parameters in multiple samples and the hyper-priors over the parameters of the prototype clusters, we constructed a hierarchical Bayesian model, FLARE. The model allows the cluster locations (given by the means) and the shapes (given by the covariance matrices) of each sample to be similar to those of their prototype cluster so that the information from multiple samples could be combined for accurate and robust estimation of clusters. At the same time, FLARE allows the clusters of each sample to be slightly different from their prototypes, accounting for the variations among different biological samples. In our experiments, we set *β*_0_ = 500 and *σ*_0_ = 6*D* so that the stochastic variation between a sample cluster mean and its prototype cluster mean is reasonable small.

Notably, in our model, a cluster can also not contain any data point in a particular sample, and thus, the cluster may be absent in certain samples. From our estimation results, we can easily distinguish which clusters are common to all samples and which only appear in certain samples.

## Variational inference

In this section, we present a variational approach to efficiently learn the approximate posterior distribution of the FLARE parameters from data.

Since there is no analytical solution to compute, the exact posterior distributions of the latent variables, a fast compilation solution is necessary. Given the large-volume of a typical cytometric data and the high-dimensionality nature of multiparametric measurements, classical Monte Carlo methods, such as Markov Chain Monte Carlo (MCMC), can be computationally costly. Thus, we resort to fast approximate Bayesian inference; in particular, we apply the Variational Bayesian method to calculate the approximate posterior distributions.

The idea of the variational inference method is to use a simpler distribution to approximate the exact posterior distribution. Specifically, the log marginal distribution can be decomposed as

$$\begin{array}{c} \ln p(X)=L(q)+KL(q,p) \end{array}$$ (9)

It consists of two parts: the lower bound L(q) and the KL divergence between *p* and *q*:

$$L(q)=\int q\left(Z\right)\;\text{ln}\;\{\frac{p(X,Z)}{q(Z)}\}dZ$$ (10)

$$KL(q,p)=-\int q\left(Z\right)\;\text{ln}\;\{\frac{p(Z|X)}{q(Z)}\}dZ$$ (11)

where *X* represents the data and *Z* represents the random variables for which we need to calculate the posterior.

The distribution *q*(**Z**) is the approximation of the true posterior *p*(**Z**|**X**). To this end, some assumptions should be exerted to the approximate posterior *q*(**Z**). One commonly used assumption is that *q* can be factorized as:

$$\begin{array}{c} q\left(Z\right)=\prod_{i=1}^{L}q_{i}\left(Z_{i}\right) \end{array}$$ (12)

Hence, by minimizing (11) given (12), we can obtain the optimized form of each factor of *q*(**Z**_*i*_) by:

$$\begin{array}{c} \text{ln}\;q_{j}\left(Z_{j}\right)=E_{i\neq j}\left[\text{ln}\;p\left(X,Z\right)\right]+\text{const} \end{array}$$ (13)

In the case of our model, we assume *q* can be factorized as

$$\begin{array}{c} q(\mu ,\Lambda ,\eta ,\Omega ,\text{h},\pi )=q(\mu )q(\eta )q(\Lambda )q(\Omega )q(h)q(\pi ) \end{array}$$ (14)

Then by using (13), we can obtain the optimized approximated posteriors as follows:

$$q({\text{h}}^{\left(m\right)})=\prod_{n=1}^{N_{m}}\prod_{k=1}^{K}{\left(r_{nk}^{\left(m\right)}\right)}^{h_{nk}^{\left(m\right)}}$$ (15)

$$q(\pi^{\left(m\right)})=C\left(\alpha^{\left(m\right)}\right)\prod_{k=1}^{K}{\left(\pi_{k}^{\left(m\right)}\right)}^{\alpha_{k}^{\left(m\right)}-1}$$ (16)

$$q(\mu_{k}^{\left(m\right)})=N(\omega_{k}^{\left(m\right)},\Gamma_{k}^{\left(m\right)})$$ (17)

$$q(\eta_{k})=N(\xi_{k},{ϒ}_{k})$$ (18)

$$q(\Lambda_{k}^{\left(m\right)})=W(\Lambda_{k}^{\left(m\right)}|\Psi_{k}^{\left(m\right)},\sigma_{m:k})$$ (19)

$$q(\Omega_{k})=W^{-1}\left(\Omega_{k}|\Phi_{k},\nu_{k}\right)$$ (20)

The parameters in these distributions are:

$$r_{nk}^{\left(m\right)}=\frac{\rho_{nk}^{\left(m\right)}}{\sum_{j=1}^{K}\rho_{nj}^{\left(m\right)}}$$ (21)

$$\begin{array}{cc} \text{ln}\;\rho_{nk}^{\left(m\right)}= & (\frac{1}{2}E[\text{ln}|\Lambda_{k}^{\left(m\right)}\left|\right]-\frac{1}{2}E\left[{\left(x_{n}^{\left(m\right)}-\mu_{k}^{\left(m\right)}\right)}^{T}\Lambda_{k}^{\left(m\right)}\left(x_{n}^{\left(m\right)}-\mu_{k}^{\left(m\right)}\right)\right]\\ & -\frac{D}{2}\text{ln}\;2\pi )+E\left[\text{ln}\;\pi_{m:k}\right] \end{array}$$ (22)

$$\alpha_{k}^{\left(m\right)}=\alpha_{0}+N_{k}^{\left(m\right)}$$ (23)

$$N_{k}^{\left(m\right)}=\sum_{n=1}^{N_{m}}r_{nk}^{\left(m\right)}$$ (24)

$$\omega_{k}^{\left(m\right)}={\left(N_{k}^{\left(m\right)}E\left[\Lambda_{k}^{\left(m\right)}\right]+\beta_{0}I\right)}^{-1}\left(N_{k}^{\left(m\right)}E\left[\Lambda_{k}^{\left(m\right)}\right]E\left[x_{k}^{\left(m\right)}\right]+\beta_{0}E\left[\eta_{k}\right]\right)$$ (25)

$$\Gamma_{k}^{\left(m\right)}={\left(N_{k}^{\left(m\right)}E\left[\Lambda_{k}^{\left(m\right)}\right]+\beta_{0}I\right)}^{-1}$$ (26)

$$\xi_{k}=\left(\epsilon_{0}\xi_{0}+\beta_{0}\sum_{m=1}^{M}E\left[\mu_{k}^{\left(m\right)}\right]\right)/\left(\epsilon_{0}+\beta_{0}M\right)$$ (27)

$${ϒ}_{k}={\left(\left(\epsilon_{0}+\beta_{0}M\right)I\right)}^{-1}$$ (28)

$$\begin{array}{ll} {\left(\Psi_{k}^{\left(m\right)}\right)}^{-1}= & \sigma_{0}E\left[\Omega_{k}^{-1}\right]+N_{k}^{\left(m\right)}(S_{k}^{\left(m\right)}+\\ & \left(E\left[{\text{x}}_{k}^{\left(m\right)}\right]-E\left[\mu_{k}^{\left(m\right)}\right]\right){\left(E\left[{\text{x}}_{k}^{\left(m\right)}\right]-E\left[\mu_{k}^{\left(m\right)}\right]\right)}^{T}) \end{array}$$ (29)

$$S_{k}^{\left(m\right)}=\left(\sum_{n=1}^{N_{m}}r_{nk}^{\left(m\right)}\left(x_{k}^{\left(m\right)}-E\left[x_{k}^{\left(m\right)}\right]\right){\left(x_{n}^{\left(m\right)}-E\left[x_{k}^{\left(m\right)}\right]\right)}^{T}\right)/N_{k}^{\left(m\right)}$$ (30)

$$\sigma_{k}^{\left(m\right)}=N_{k}^{\left(m\right)}+\sigma_{0}$$ (31)

$$\Phi_{k}=\Phi_{0}+\sigma_{0}\sum_{m=1}^{M}E\left[\Lambda_{k}^{\left(m\right)}\right]$$ (32)

$$\nu_{k}=\nu_{0}+M\sigma_{0}$$ (33)

And the expectations in the above equations are:

$$E[x_{k}^{\left(m\right)}]=\sum_{n=1}^{N_{m}}r_{nk}^{\left(m\right)}x_{n}^{\left(m\right)}/N_{k}^{\left(m\right)}$$ (34)

$$E[\text{ln}|\Lambda_{k}^{\left(m\right)}|]=\sum_{i=1}^{D}\psi (\frac{\sigma_{k}^{\left(m\right)}+1-i}{2})+D\;\text{ln}\;2+\text{ln}\left|\Psi_{k}^{\left(m\right)}\right|$$ (35)

$$\begin{array}{c} E[{\left(x_{n}^{\left(m\right)}-\mu_{k}^{\left(m\right)}\right)}^{T}\Lambda_{k}^{\left(m\right)}\left(x_{n}^{\left(m\right)}-\mu_{k}^{\left(m\right)}\right)]\\ =D/\beta_{0}+\sigma_{k}^{\left(m\right)}{\left(x_{n}^{\left(m\right)}-\omega_{k}^{\left(m\right)}\right)}^{T}\Psi_{k}^{\left(m\right)}\left(x_{n}^{\left(m\right)}-\omega_{k}^{\left(m\right)}\right)+tr\left(\Gamma_{k}^{\left(m\right)}\Psi_{k}^{\left(m\right)}\right) \end{array}$$ (36)

$$E[\text{ln}\;\pi_{k}^{\left(m\right)}]=\psi \left(\alpha_{k}^{\left(m\right)}\right)-\psi \left(\sum_{j=1}^{K}\alpha_{j}^{\left(m\right)}\right)$$ (37)

$$E[\Lambda_{k}^{\left(m\right)}]=\sigma_{k}^{\left(m\right)}\Psi_{k}^{\left(m\right)}$$ (38)

$$\begin{array}{c} E\left[\eta_{k}\right]=\xi_{k},\;\;E\left[\mu_{k}^{\left(m\right)}\right]=\omega_{k}^{\left(m\right)},\;\;E\left[\Omega_{k}^{-1}\right]=\Phi_{k}^{-1}\nu_{k} \end{array}$$ (39)

where *ψ*(⋅) is the *digamma* function—the logarithmic derivative of the gamma function $\psi \left(x\right)=\frac{d}{dx}\;\text{ln}\;\Gamma \left(x\right)=\Gamma^{\prime }\left(x\right)/\Gamma \left(x\right)$.

## Parallel inference

Thousands of cells are processed by a flow cytometer every second, which results in an extremely high volume of data to analyze. For example, one of our data sets contains nearly 2.6 million cells spread across 14 samples. Due to the complexity of our model and the size of flow cytometry data, there is a critical need to develop a parallel algorithm which can take advantage of the processing power in a large-scale computer cluster. While the sequential version of FLARE was implemented using MATLAB, the parallel version is implemented using C++ and MPI.

A computer cluster consists of many separate nodes, i.e. computers, connected via a fast local area network. Additionally, each node may contain a multi-core processor. This allows us to devise a two-level parallelization scheme to analyze the data. At the first level of parallelization, we divide the data amongst the cluster nodes. Hence, each cluster node is responsible for a portion of the raw data as well as maintaining any parameters associated with that data. For example, all sample means (***ω***) and sample precision matrices (**Ψ**) for sample 1 need to be stored on any cluster node which contains data from sample 1. In an effort to minimize repeated storage, we impose the restriction that each node must only store data from a single sample. Additionally, every cluster node may have a multi-core processor, which enables us to implement a second level of parallelization based on the number Gaussian components (*K*). Many of the parameters we infer are indexed by *k*, e.g. the prototype mean ***ξ*** is really a set of *K* prototype means (one mean for each component). Therefore, we can use the set of processor cores to optimize the variational inference parameters for each value of *k* in parallel.

### Data partition

Each iteration of the parallel inference algorithm alternates several times between computation and communication phases. All nodes must complete their current computation phase before the next one one can begin. Therefore, the total execution time is dependent on the node with the highest computational load (the node that take the longest). The goal of load balancing is to minimize the largest computational load of the nodes in the computer cluster.

Suppose we have a computer cluster consisting of a total of *W* nodes. We must now find some way to distribute the data among these *W* nodes that minimizes the total execution time. A naive approach would be to evenly divide the *N* data points so that each node is responsible for *N*/*W* data points. However, the volume of data assigned to each node is not the only factor that influences computation time. A node must also maintain all parameters associated with its data. For instance, a node with data from samples 1 and 2 will need to maintain means and precision matrices for both of these samples, whereas a node that only has data from sample 1 will maintain means and precision matrices for sample 1 only. Therefore the time spent spent optimizing distribution parameters can be reduced by restricting each node to data from a single sample.

We can think of the balancing problem in this way: we have *W* nodes available, and a subset of these nodes (*W*_1_) must be assigned to sample 1, another subset (*W*_2_) must be assigned to sample 2, and so on for all *M* samples. The data of a particular sample is divided evenly among the nodes assigned that sample. The load for each node assigned to a particular sample is equal to *N*_*m*_/*W*_*m*_. Algorithm 1 gives us a greedy strategy to minimize the load on the sample with the largest load. In order for this algorithm to function correctly, we must declare a larger number of nodes than there are samples.

**Algorithm 1** Balance the computation load across the available cluster nodes

1: **function** NodeBalance

2:  **if** The number of nodes is less than the number of samples **then**

3:   Error!

4:  **end if**

5:  Assign one node to each sample.

6:  **while** There are unassigned nodes **do**

7:   Assign a node to the sample with the highest load.

8:  **end while**

9: **end function**

We define the data partition efficiency by

$$\begin{array}{c} \text{balance}=\frac{{\text{load}}_{\text{opt}}}{{\text{load}}_{\text{max}}} \end{array}$$ (40)

Since the optimum balance would have the same computational load on each node, we define load_opt_ by

$$\begin{array}{c} {\text{load}}_{\text{opt}}=\left\lceil W/N\right\rceil \end{array}$$ (41)

Where

$$\begin{array}{c} N=\sum_{m=1}^{M}N_{m} \end{array}$$ (42)

Also, we define the maximum load (load_max_) by

$$\begin{array}{c} {\text{load}}_{\text{max}}=\underset{m=1,‥,M}{\text{argmax}}\left(W_{m}\right) \end{array}$$ (43)

We know that load_max_ ≤ load_opt_ because any time the load differs from the optimum, we must have some node with a larger load than load_opt_, and some other node with load smaller than load_opt_. Therefore, an optimally balanced set of nodes will give us a data partition efficiency score of 1, and any non-optimally balanced set of nodes will give us a score less than 1. Also since the true computational cost is dependent on the slowest node, we use the node with the largest load to define the data partition efficiency.

### Organization of parameters across cluster nodes

The raw data is not the only information we must store. The variational inference method gives us a set of parameters we must iteratively optimize. Namely, these parameters are **r**, ***ρ***, *α*, *N*, ***ω***, *E*[**x**], **Γ**, ***ξ***, **Ψ**, ***S***, *σ*, **Φ**, and *ν*. We can divide these parameters into 3 separate categories based of how they are indexed. The first category includes all parameters indexed by sample and by data point. These parameters include *r*, *ρ*, and the raw data *x*. The second category includes all parameters indexed by sample. These parameters include *α*, *N*, ***ω***, *E*[**x**], **Γ**, **Ψ**, and ***S***. The third category includes all parameters which are not indexed by sample or by data point. These parameters include ***ξ***, **Φ**, and *ν*. To show how these three groups of parameters are stored on the cluster, we define three new parameters, *A*, *B*, and *C*.

*A* is used to represent the first category and is indexed in the following way. *A*’s superscript is indexed by sample, so *A*^(*m*)^ includes *r*^(*m*)^, *ρ*^(*m*)^, and **x**^(*m*)^ for all *m* = 1, …, *M*. Furthermore, each *A*^(*m*)^ is split up into *W*_*m*_ different parts, where *W*_*m*_ is the number of nodes assigned to sample *m*. Each of these *W*_*m*_ parts contains an equal portion of the *N*_*m*_ data points in sample *m*. So, $A_{1}^{\left(m\right)}$ includes $r_{1}^{\left(m\right)}$ to $r_{d_{m}}^{\left(m\right)}$, $\rho_{1}^{\left(m\right)}$ to $\rho_{d_{m}}^{\left(m\right)}$, and $x_{1}^{\left(m\right)}$ to $x_{d_{m}}^{\left(m\right)}$, where *d*_*m*_ = *N*_*m*_/*W*_*m*_. Similarly $A_{2}^{\left(m\right)}$ contains the next *N*_*m*_/*W*_*m*_ indices of *r*^(*m*)^, *ρ*^(*m*)^, and **x**^(*m*)^, and so on for all *W*_*m*_ portions.

The topographies of *B* and *C* are much simpler. *B* is used to represent the second category and is indexed by sample. Hence, *B*^(*m*)^ includes *α*^(*m*)^, *N*^(*m*)^, ***ω***^(*m*)^, *E*[**x**], **Γ**^(*m*)^, **Ψ**^(*m*)^, and *S*^(*m*)^ for all *m* = 1, …, *M*. *C* is used to represent the third category and is not indexed.

**Fig 2:**
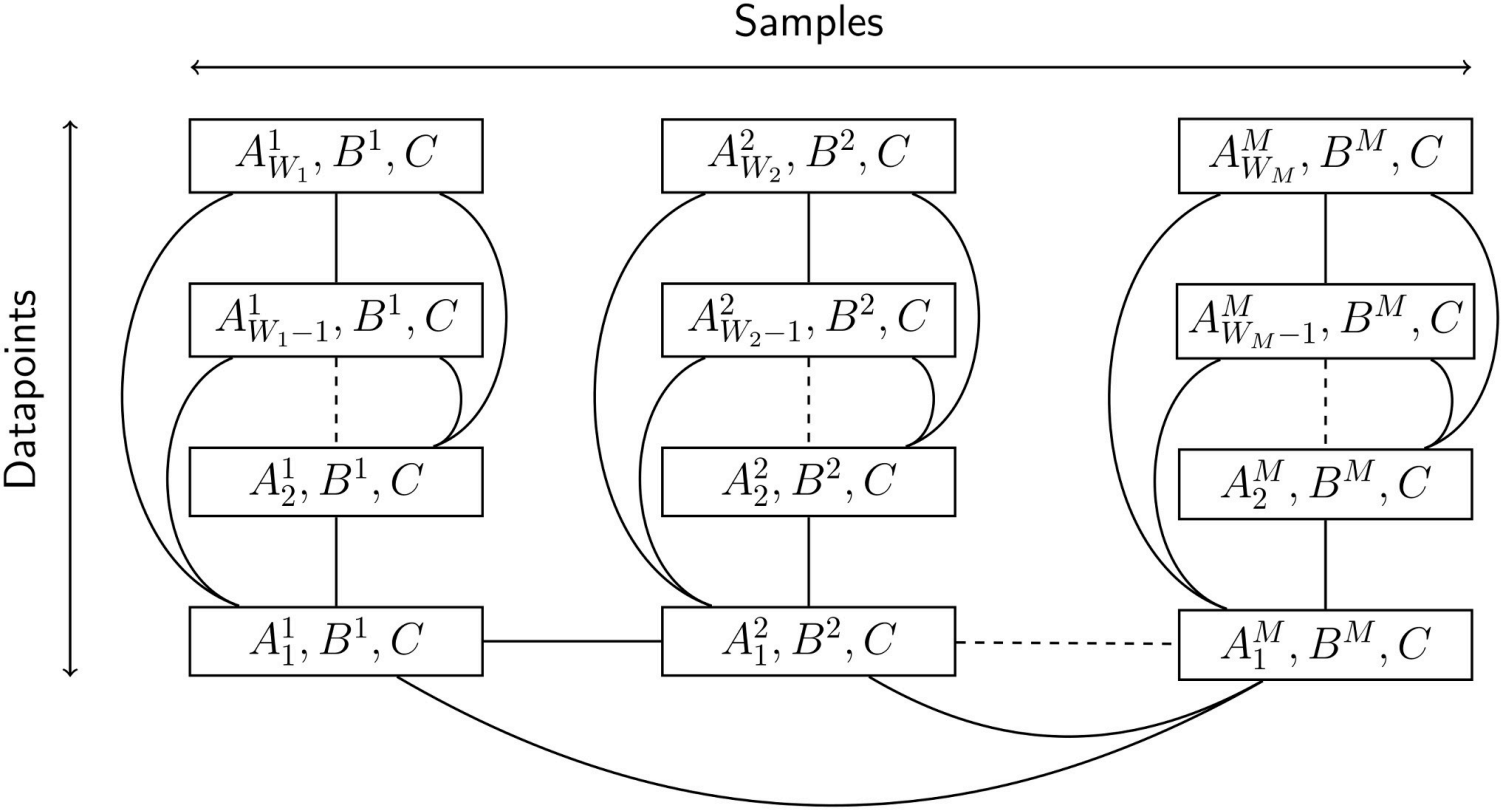
Topography of data storage among cluster nodes. Each rectangle represents a cluster node, with each column consisting of nodes from a particular sample. The edges of this graph connect nodes which must communicate with each other. The nodes of each column form a fully connected subgraph to show the communication done within each sample. Similarly, each column has representative node that participates in summations over all samples. The dotted edges represent the fact that based on the data, there can be an arbitrary number of samples and nodes per sample.

With the parameters *A*, *B*, and *C* in hand, we can visualize the overall cluster topography as shown in Fig 2. Using this topography, the calculations of Eqs (21) and (22) are split up among every node with no repeated calculation. The calculation of Eqs (23), (25), (26), and (31) can be done with no communication. However the calculation of these equations is repeated on every node assigned to a particular sample, e.g. the calculation of these equations for sample 1 is repeated on all nodes assigned to sample 1. Eqs (24), (34), and (30) all involve a summation indexed from 1 to *N*_*m*_. The nodes of sample *m* all calculate their partial sum using their portion of the data, then communicate to calculate the total sum. The calculations for each sample can be done simultaneously. Lastly, Eqs (27) and (32) involve a summation indexed from 1 to *M*. To perform this calculation a representative node from each sample is chosen to contribute its partial sum. Each of these representative nodes then communicate their results to the rest of the nodes assigned to their respective samples.

### Further parallelization using p-threads

Each cluster node may have a multi-core processor. With the exception of Eq (21), each of the parameter update equations for variational inference are indexed by Gaussian components, where each *k* = 1, …, *K* is independent. Therefore, all of these equations may be updated simultaneously using p-threads.

## Results and discussion

**Fig 3:**
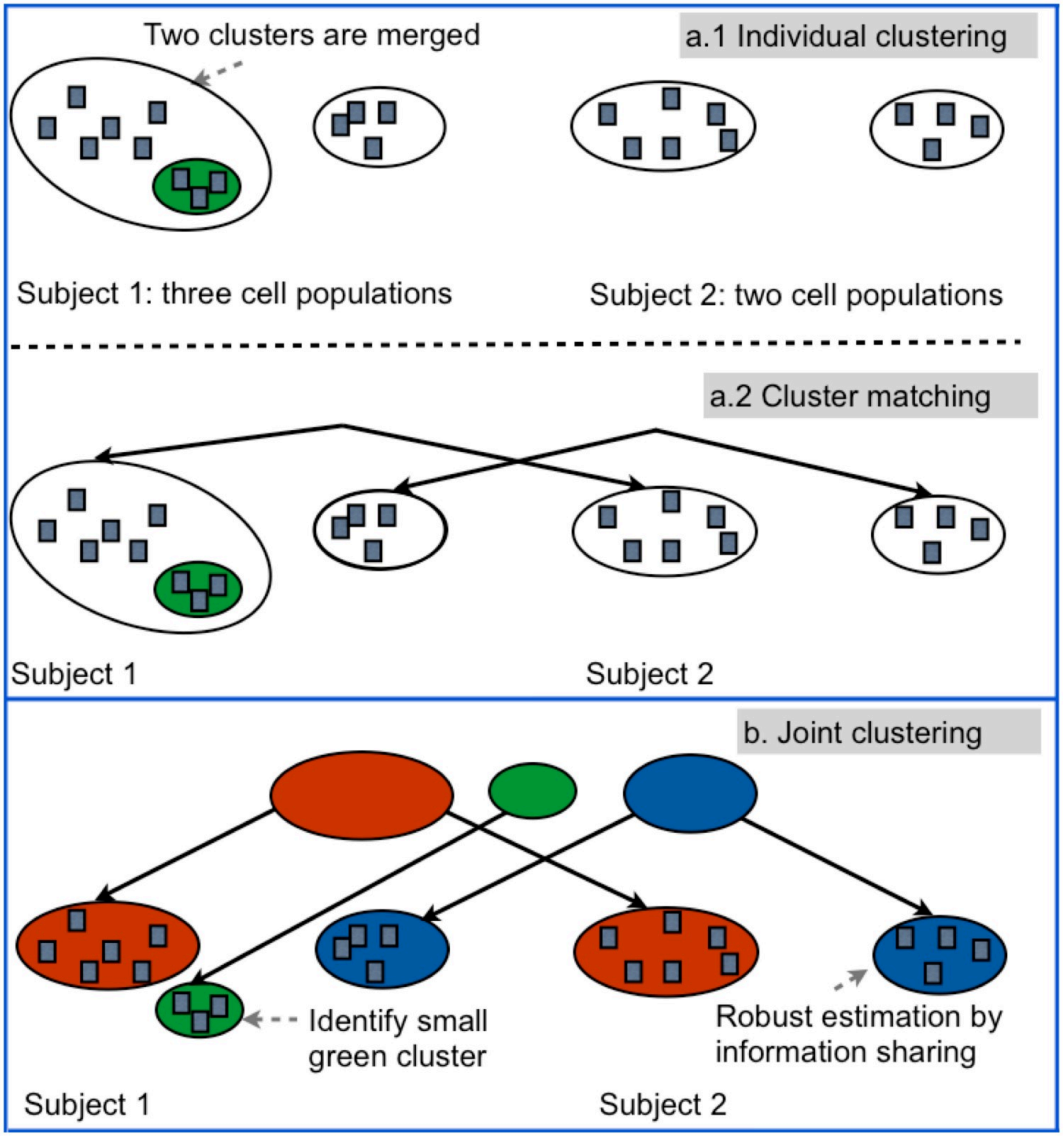
Illustrative example for FLARE and its graphical model representation. Panels a.1 and a.2 show the limitation of separate population analysis on individual samples: it misses the detection of the rare cell population in green. Panel b shows that by sharing population information via the parental nodes, FLARE can more accurately estimate the big cell population in the red cluster and also detect the rare population in the green cluster. Panel c describes the hierarchical Bayesian model of FLARE.

We developed a new computational framework FLARE for FLow cytometric Analysis of Rare Events, although it may be applicable to other platforms that generate multi-marker data per cell. FLARE is based on a hierarchical Bayesian model, and employs parallel computation for its high-speed high-precision analysis. The Bayesian model (Fig 3) of FLARE allows implementation of several distinct features to specifically address the challenges mentioned above. For consistent identification of a particular rare population *C*, the model parameters allow information about *C* to be shared across different samples. In our parallel computing framework, we implemented this via communication among nodes each of which analyzed a distinct sample. The strategy builds repeated inter-sample consensus on the existence of *C* (or lack thereof), thus guarding against unsupervised detection of possibly numerous spurious small populations. Consequently, the model estimation is robust against high inter-sample variation and platform noise, which otherwise are known to affect the reproducibility or the quality of match between analogous populations across samples and replicates [9].

We found FLARE’s information-sharing feature to be especially useful for rare cell populations (e.g., blue clusters in Fig 3) which contain very few cells since it effectively pools together more observations for estimation. Second, the estimation ambiguity (between the red and the green clusters in Sample 1, corresponding to Subject 1, in Fig 3) is reduced since the information about a population (e.g., the red cluster of Sample 2, corresponding to Subject 2, in Fig 3) can guide the estimation of its counterpart across samples (i.e., the red cluster of Sample 1 in Fig 3) in FLARE’s joint model. Third, the joint model also allows partial consistency such that some clusters can exist in one or more samples but not necessarily in all of them. Thus, without needing any additional cluster alignment [4, 10], we can identify also those clusters (e.g., the green cluster in Fig 3b) that exist only in certain samples, a situation that is not uncommon for rare populations e.g., transient subsets that are present only during certain stages of cell differentiation and are absent otherwise).

For our first application of FLARE, we generated a 6-marker cytometric dataset to study cells from mouse bone-marrow. These murine studies of normal hematopoietic stem and progenitor cells were conducted under an IACUC (Institutional Animal Care & Use Committee) approved protocol at Yale University. Mice were euthanized following Yale IACUC recommendation using carbon dioxide.

**Fig 4:**
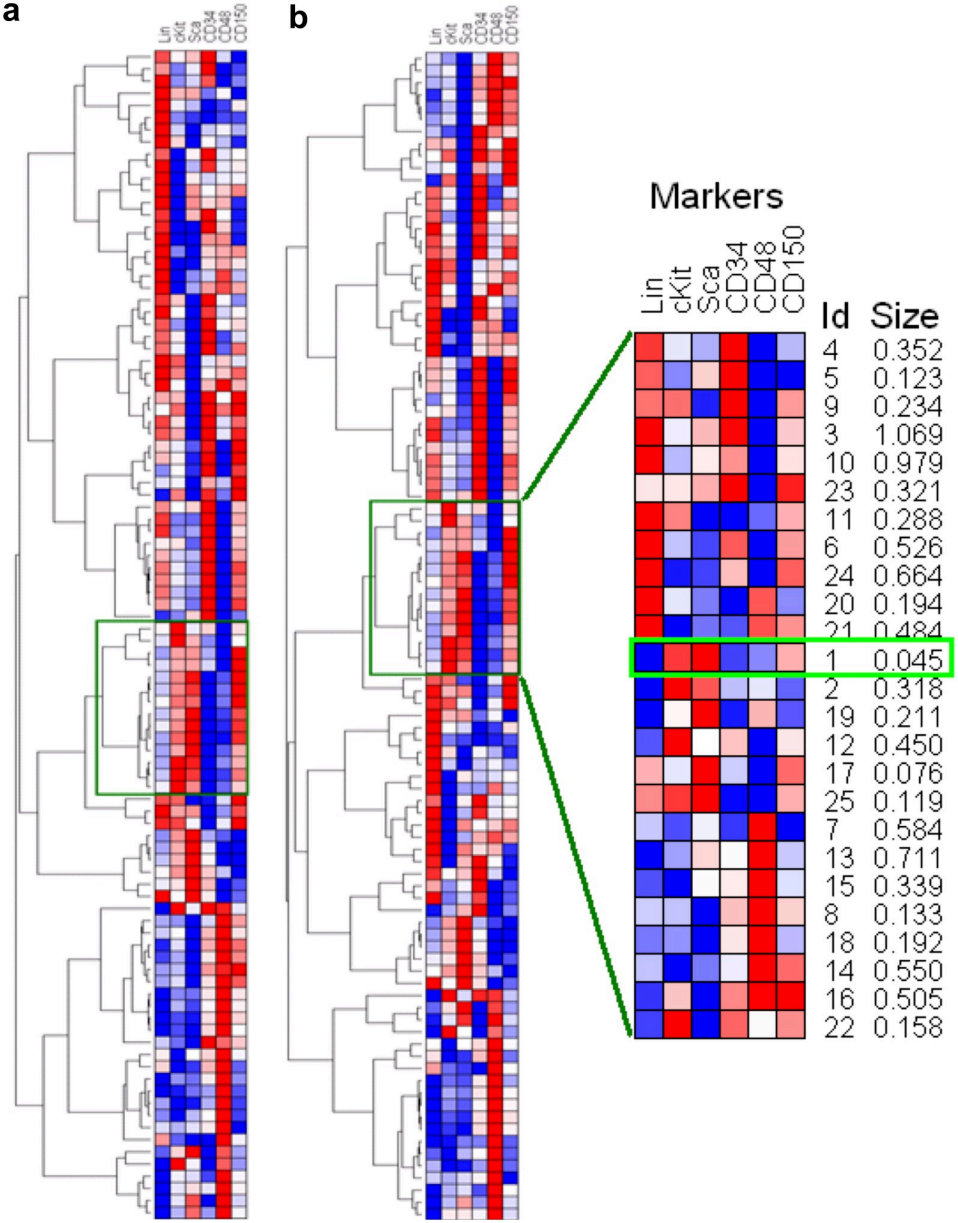
FLARE identifies rare HSC population in murine bone marrow samples undergoing normal hematopoiesis. (a) In the first step, unsupervised clustering by the hierarchical Bayesian model is used to explore the different subsets in 6-dimensional marker space. In 12 blood samples (3 biological replicate mice, 4 technical replicates per specimen), FLARE identified 96 populations matched across all samples. Plots (a) and (b) show the heatmaps for two representative samples, where each row represents a population’s mean intensities for the 6 markers represented by the columns. Red/blue is used to depict high/low intensities. Thus a common region of interest, shown in green rectangle, was identified. In the 2nd step, in a new and much larger sample, FLARE zoomed into the specified region to detect the clusters therein. The uncovered hierarchy of populations is shown with a heatmap in plot (zoomed in right panel). We identified one particular population (denoted by cluster #1; light green rectangle) that has the size (0.045%) and marker-signature (Lineage^−^c-kit^+^Sca^+^CD34^-^CD48^-^CD150^hi^) consistent with the LT-HSC cells.

In the first step, without any human guidance, unsupervised analysis by FLARE was run on 14 “training” samples, including multiple biological and technical replicates, and it identified a subset bearing a 6-marker signature of long-term murine hematopoietic stem cells (LT-HSCs) (Fig 4a and 4b). In the second step, using this signature location parameter, FLARE focused on the corresponding region in an entirely new and much larger “test” sample (containing more than a million cells measured with the same 6 markers) to detect a very rare population (containing 0.045% of the total number of cells in the sample) with a more precise LT-HSC signature. This finding is supported by earlier analyses using sequential two-dimensional gating, according to which LT-HSCs are known to be Lineage^−^Kit^+^Sca^+^CD34^−^CD48^−^CD150^+^. Using parallel computation, the two steps took less than 10 minutes to finish. The consistency of the detected subsets across all 14 training samples, the small size of the final detected population and the precise marker-expressions of the cells therein all demonstrate how FLARE could be used for high-speed automated identification of rare populations in cytometric data.

**Fig 5:**
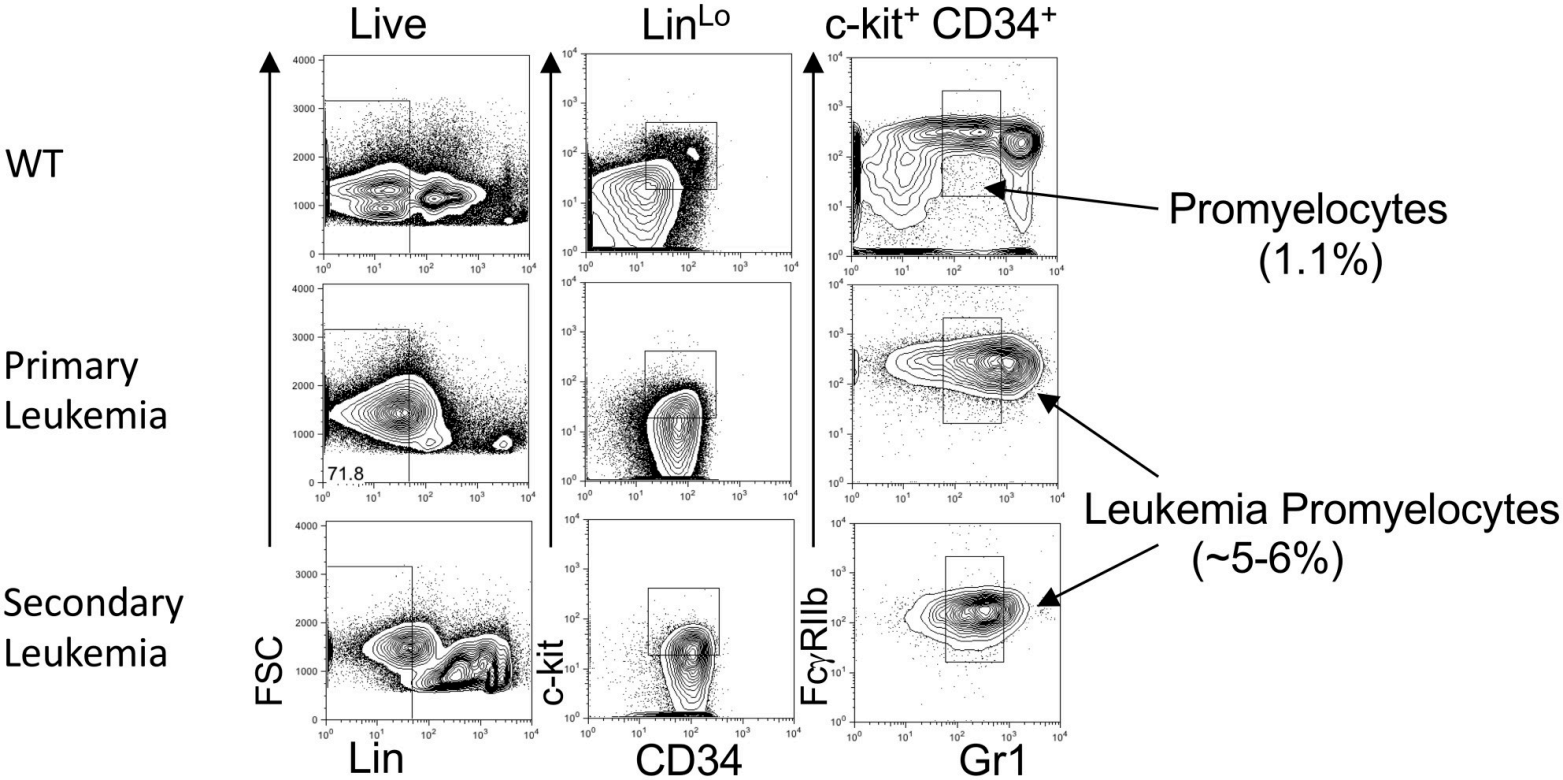
FLARE in PML-RAR*α* transgenic mouse model. Wildtype (C57BL6) bone marrow, primary leukemic mice with PML-RAR*α* transgenic and secondarily transplanted mice were analyzed by flow cytometry (and gated as previously described [12]). Forward scatter (FSC), Lineage staining (Lin) are gated serially from left panels to right. Experimentally determined surface phenotype of leukemic promyleocytes are gated (right panel), and frequency of this population is shown among live cells.

As a second application of FLARE, we used it for identifying a rare signature of a disease during its progression. For this purpose, we used a model of myeloid leukemia that harbors the oncogenic fusion of the PML gene and the retinoic acid receptor alpha (RAR*α*). These mice succumb to a lethal acute promeylocytic leukemia (APL) that can be subsequently transplanted with increasing aggressiveness [11]. We have previously characterized the cell surface phenotype which drives the APL and it closely resembles the normal promyelocyte population [12, 13]. Notably, the mature granulocytes, which differentiate from the leukemic stem cell population (LSC) are unable to transplant the disease. The manual gating strategy for this population is challenging since these cells express low levels of lineage markers and are sequentially gated for CD34/c-Kit and then Gr1/ FcgRIIb (Lin^lo^ c-kit^+^ CD34^hi^ Gr^mid^ FcgRIIb^+^). This population in a normal bone marrow is approximately 1% of the live bone marrow cells and increases to 5-6% in the leukemic mice (Fig 5a).

**Fig 6:**
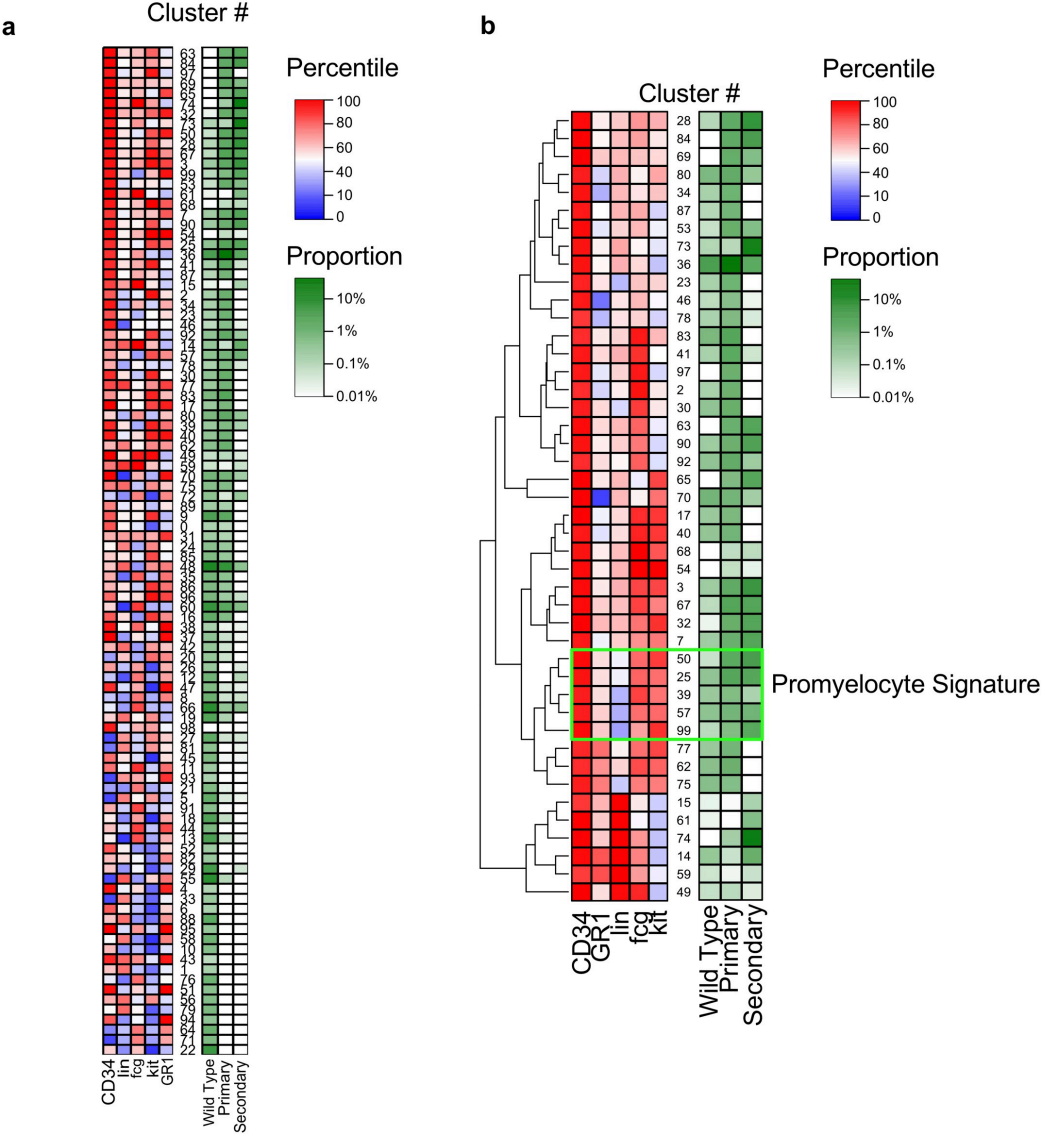
FLARE in PML-RAR*α* transgenic mouse model. Flow cytometric data from mice in panel (a) were analyzed with FLARE and a heat map of all 99 clusters with a surface phenotype and the proportion of the particular clusters among the live cells. The populations with greater than 1-fold change in their proportions from primary or secondary leukemia compared to wild type bone marrow cells are shown in panel (b). Green box indicates clusters (50, 25, 39, 57 and 99) that contain the previously experimentally derived known surface phenotype of the leukemic promyelocytes (Lin^lo^CD34^hi^c-Kit^hi^Gr1^mid^FcgRIIb^hi^). The left panel represents the percentile of each flow staining parameter ranked among the 99 clusters. The right panel indicates proportion of live-gated cells within each cluster.

By running FLARE on live-gated cells stained with markers for lineage, c-kit, CD34, Gr-1 and FcgRIIb we could identify 99 unique clusters that contained cells (Fig 6a). To determine if the found clusters contained the LSC population, we focused on the populations with greater than 1-fold change in their proportions compared to the normal mice. This allowed us to “zoom” into 44 clusters of hematopoietic cells that increased during leukemia progression which were visualized with a heatmap (Fig 5b). We identified a block of clusters that matched with the known surface phenotype of the promyelocytes and the LSCs, which we named the “Promyelocyte Signature” (Clusters 99, 57, 39, 25, 50 in the green box, Fig 3c). We found the promyelocyte cluster to represent 1% of the live cells in the normal mice, which increased to 6-8% in the leukemic mice (Fig 6b). FLARE successfully identified this rare population and demonstrates the utility for tracking changes within phenotypically defined populations during disease progression. Using parallel computing, this was accomplished in under 5 minutes.

The stochastic expression of cells in high-dimensional marker-space of cytometric data naturally leads to the idea of modeling each cell population with a multivariate statistical distribution whose parameters can describe its characteristics [14]. Over the past decade, computational cytometric studies have therefore led to a number of new applications of finite mixture models [4, 6, 15–17]. Some of these have also involved hierarchical and multi-level models [8, 10, 17]. Often, such methods were designed with the aim of detecting both known as well as rare cell clusters [4, 18–20] in an automated manner. Other studies have developed fast clustering algorithms with the aim of handling large cytometric data [21–23]. FLARE combines the merits of such methods and aims and uses the power of parallel computation to provide the unique means of sharing information across and during the fitting to each sample an overall hierarchical mixture model while allowing for sample-specific variations.

FLARE offers several distinct advantages specifically for characterization of rare populations. First, FLARE shares information across multiple samples in a hierarchical Bayesian model (Fig 1) to identify cell populations in all samples or in only part of samples. Unlike common clustering methods, FLARE does not need a priori specification of the optimal number of clusters in data, which gives it an advantage while searching samples which may contain populations ranging from significantly big to extremely rare. Instead, FLARE automatically allows an initial mixture model with a large number of components to become sparse as the inconsistent clusters are removed and the actual number of clusters used to fit the data is learned in the process. In practice, FLARE can be viewed as an efficient approximation to Dirichlet Process Mixture (DPM) models, which have been used in the past [10]. Of course, FLARE uses the strategy of information sharing for fitting robust models by verifying the rare clusters across samples. Simulation results showed that FLARE achieves favorable estimation performance over alternative methods (S1–S4 Figs).

Further, FLARE is fit with a Variational Bayes approach which provides computationally efficient and accurate estimation of all the latent variables, i.e., the output of the model. Furthermore, FLARE modeling is parallelized with careful consideration on workload balancing in a distributed computing environment. It achieves almost linear speedup given more computational nodes (S5 Fig), making it truly scalable for large datasets.

Identification of rare cell subsets—while establishing their correspondence across multiple samples—can (a) reveal, in an unsupervised way, the overall structure among the populations, both big and small, with respect to each other in every sample, and thereby (b) provide contextual information that helps in supervised dissection of the chosen regions of interest in the marker-space to characterize the rare populations with further precision. Such progressive “zooming in” capability of FLARE mimics the strength of sequential manual gating. An important advantage of FLARE’s Bayesian design is that it can be made to systematically zoom into interesting regions or populations by a priori specifications. Thus FLARE can perform increasingly finer clustering using the same mathematical model, which can again match and verify the finer subpopulations across multiple samples. This allows FLARE to combine the benefits of an unsupervised clustering method with supervised analysis of manual sequential gating. We illustrated these aspects of FLARE using a multi-step analysis of a hierarchy of cell populations as observed in two datasets based on (i) normal hematopoiesis in mice, and (ii) oncogenic progression in a mouse model. Further examples of FLARE analyses of secondary (Treg) and simulated datasets along with the performance results are described in S1 File and S1–S5 Figs.

## Conclusion

In summary, the hierarchical design and distributed variational estimation allows FLARE to share information about corresponding clusters across samples, and quickly detect a variety of populations, including considerably rare ones, in an unsupervised manner. In the process, it efficiently searches the high-dimensional marker-space to reveal the underlying population structure. Thereupon it can progressively concentrate its search within regions of interest and also perform supervised analysis of subpopulations similar in principle to manual gating except FLARE does it in high-dimensions and with mathematical rigor. In our future work we look forward to embedding this step into FACS systems for real-time sorting of the desired cells. Since the multi-parametric population signatures reported by FLARE are quantitative and precise, however rare the underlying events may be, it helps to verify and eventually standardize definitions of specific cellular species, allow objective extraction, and facilitate reproducible cytometric analysis. Finally, the parallel estimation algorithm in FLARE is currently implemented using Message Passing Interface (MPI) and can be readily adapted to popular distributed computing platforms.

## Supporting information

S1 Fig The adjusted rand index of each method on the synthetic datasets. We use the hard clustering results of the subject who has the small clusters to compute the ARIs against true clustering assignment.
(PDF)

S2 Fig Visualization of clustering results in synthetic data. (PDF)

S3 Fig Maximum Jaccard index on Treg dataset. (PDF)

S4 Fig Maximum detection accuracy on Treg dataset. (PDF)

S5 Fig Speedup rate and load balancing efficiency. The top panel shows the speedup rate of our parallel inference algorithm using increasingly more cluster nodes. The bottom panel shows the load balancing efficiency. The balancing efficiency is calculated using Eq (32). With more nodes, the data are more evenly distributed so that the balancing efficiency keeps increasing.
(PDF)

S1 File Supplemental materials for ‘high-speed automatic characterization of rare events in flow cytometric data’. Further details on the Experimental Results.
(PDF)
