# Supplementary figures and images for: High-speed automatic characterization of rare events in flow cytometric data
Source: PLoS One. 2020 Feb 11;15(2):e0228651. doi: 10.1371/journal.pone.0228651 (PMC7012421; doi:10.1371/journal.pone.0228651)

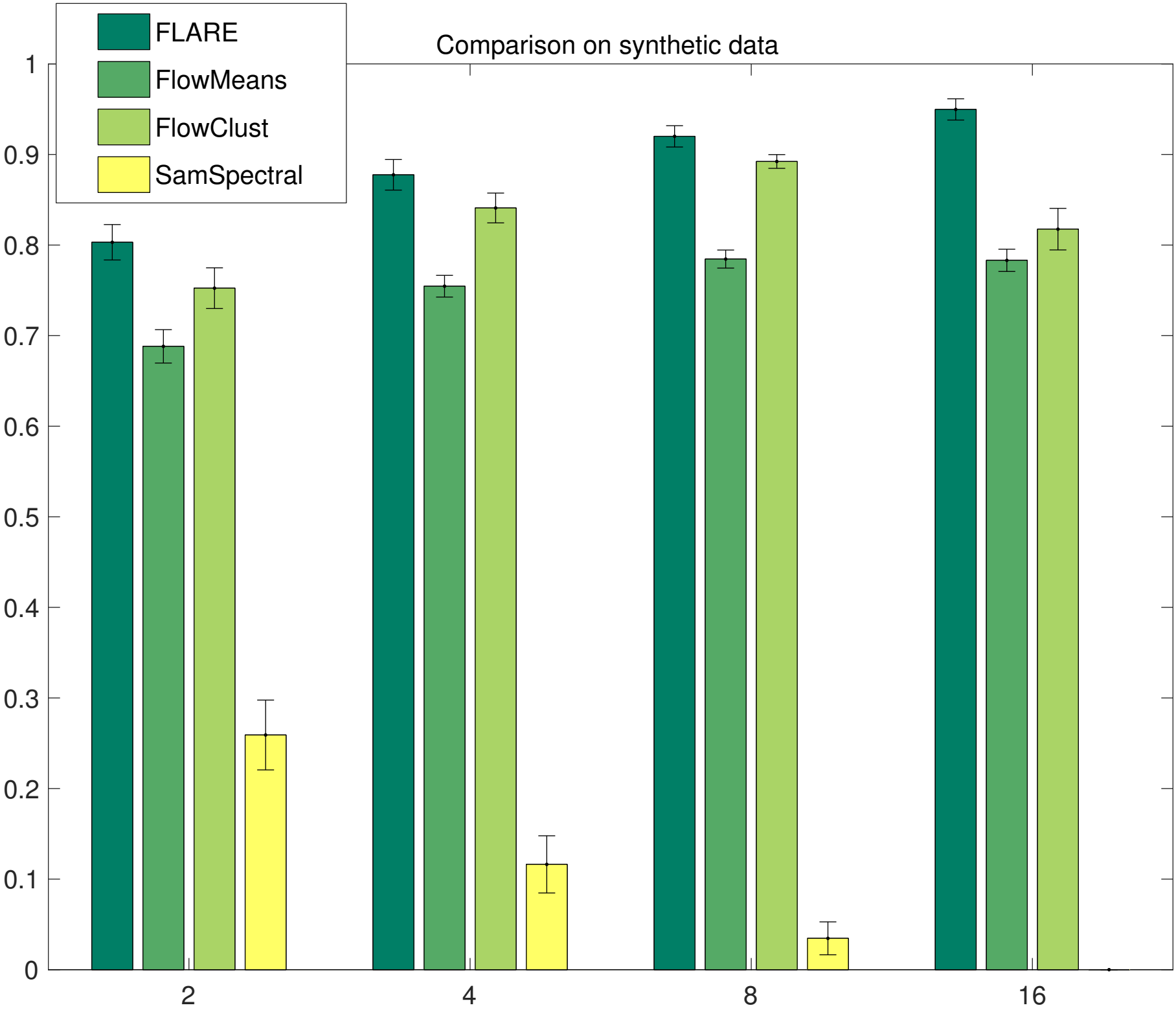

Supplement: S1 Fig — We use the hard clustering results of the subject who has the small clusters to compute the ARIs against true clustering assignment. (PDF) [file pone.0228651.s001.pdf]

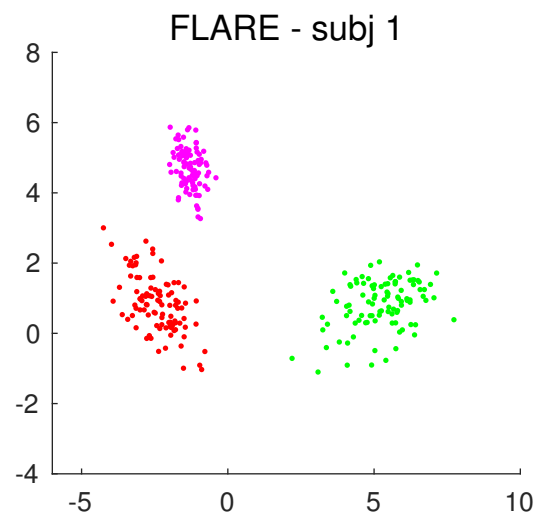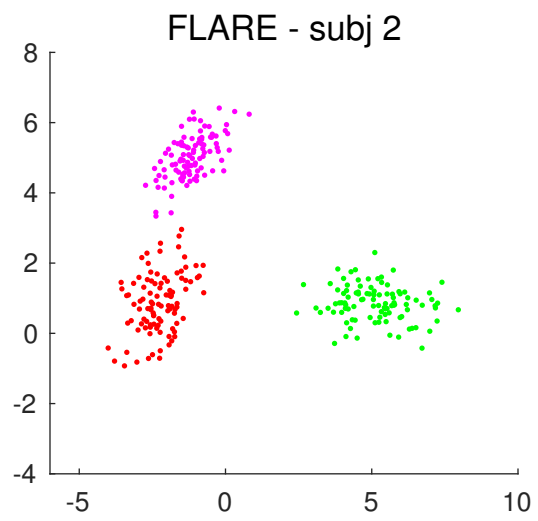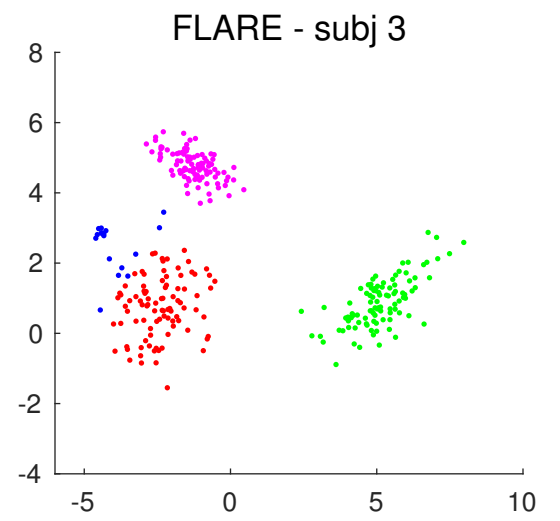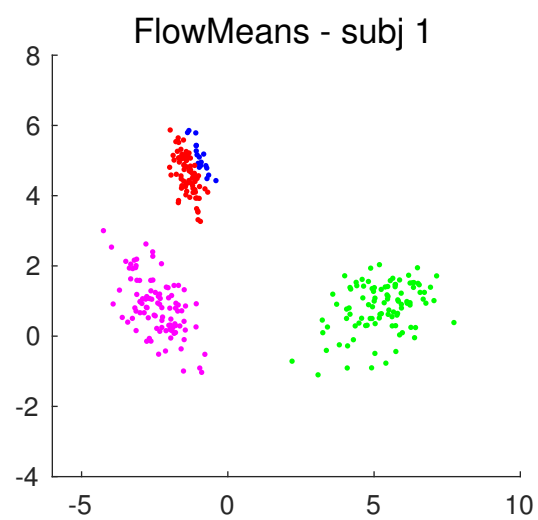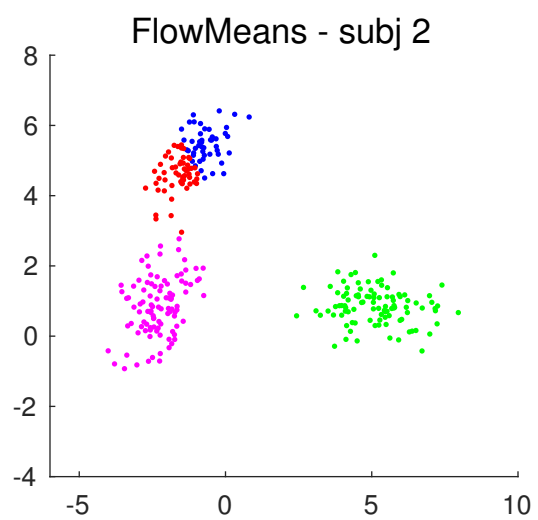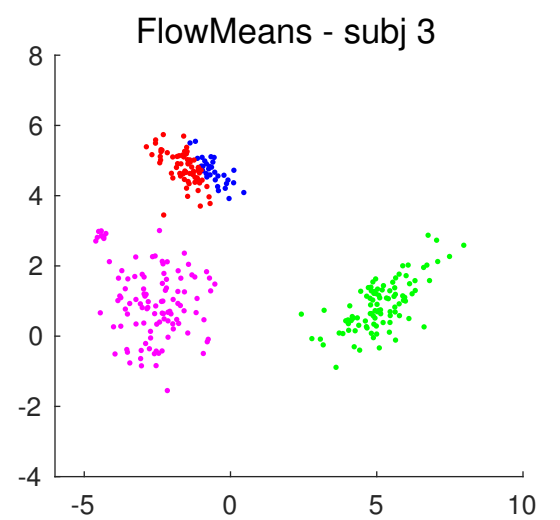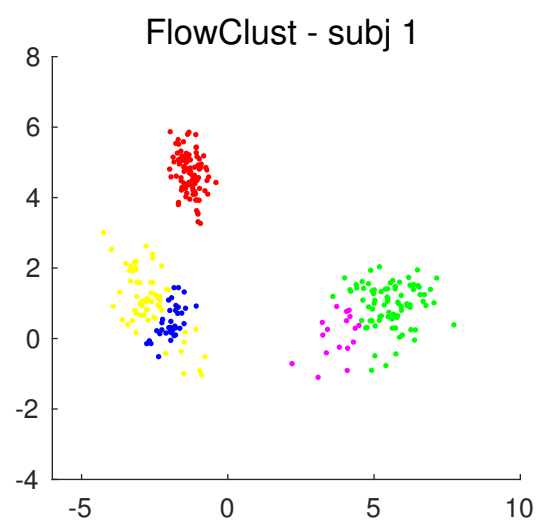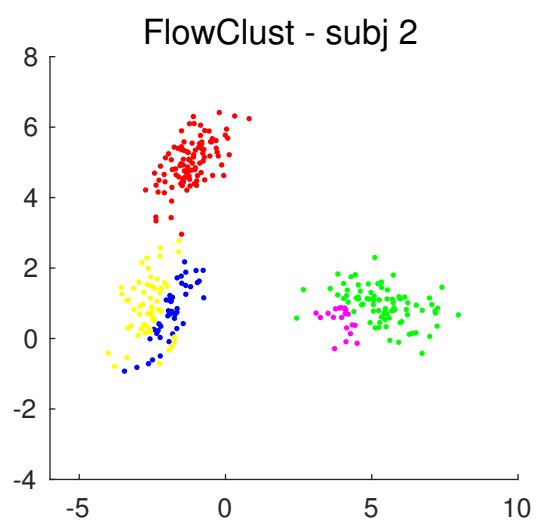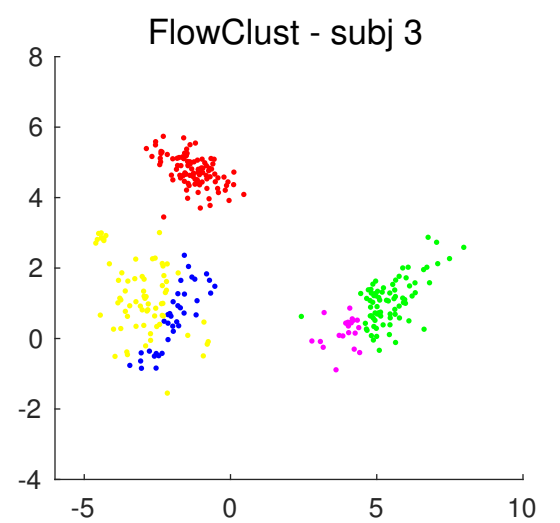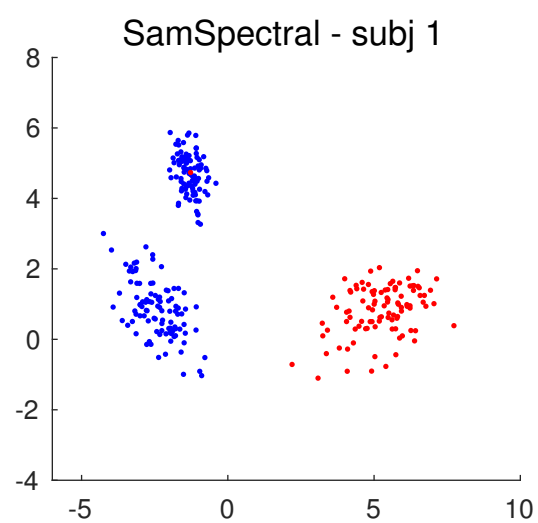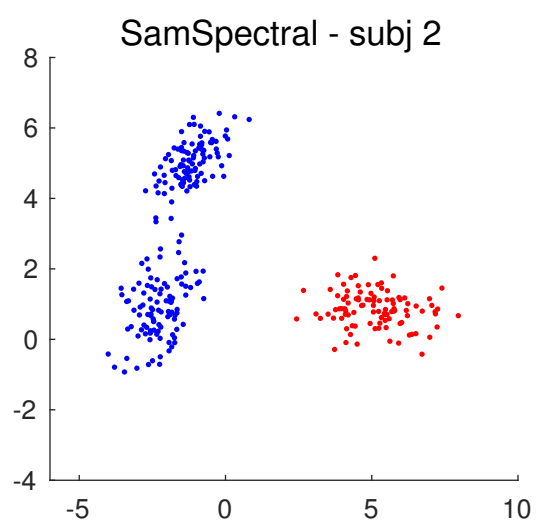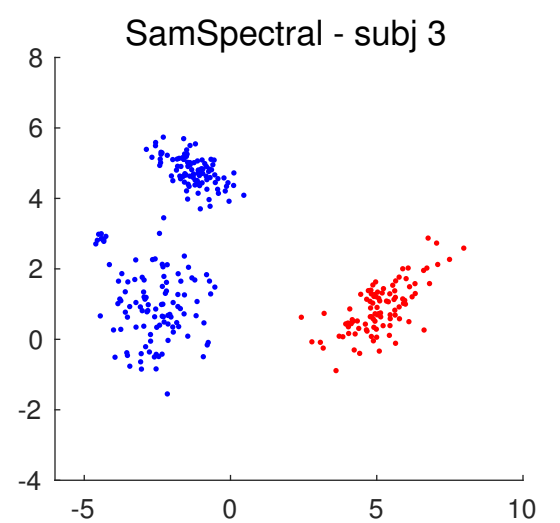

Supplement: S2 Fig — (PDF) [file pone.0228651.s002.pdf]

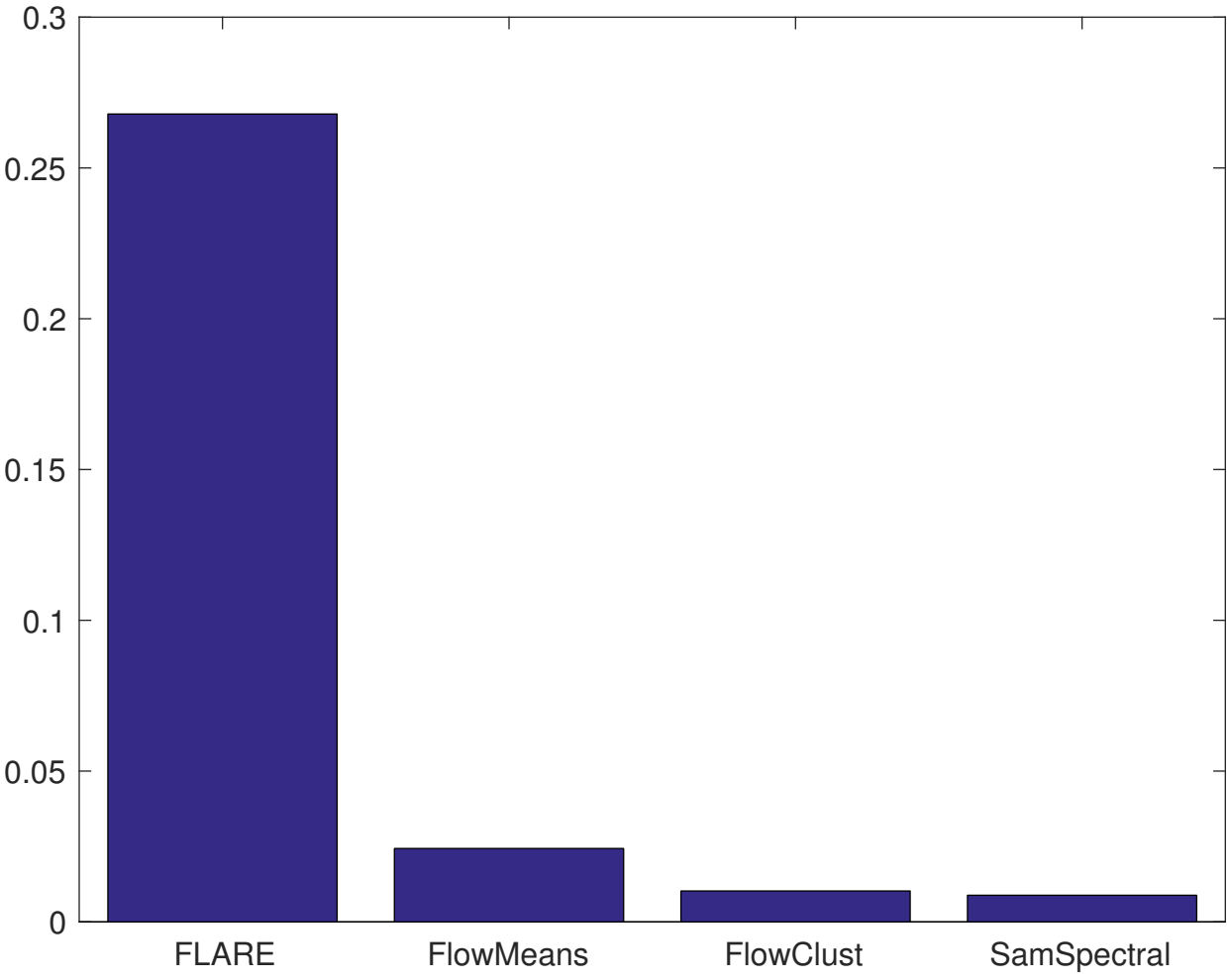

Supplement: S3 Fig — (PDF) [file pone.0228651.s003.pdf]

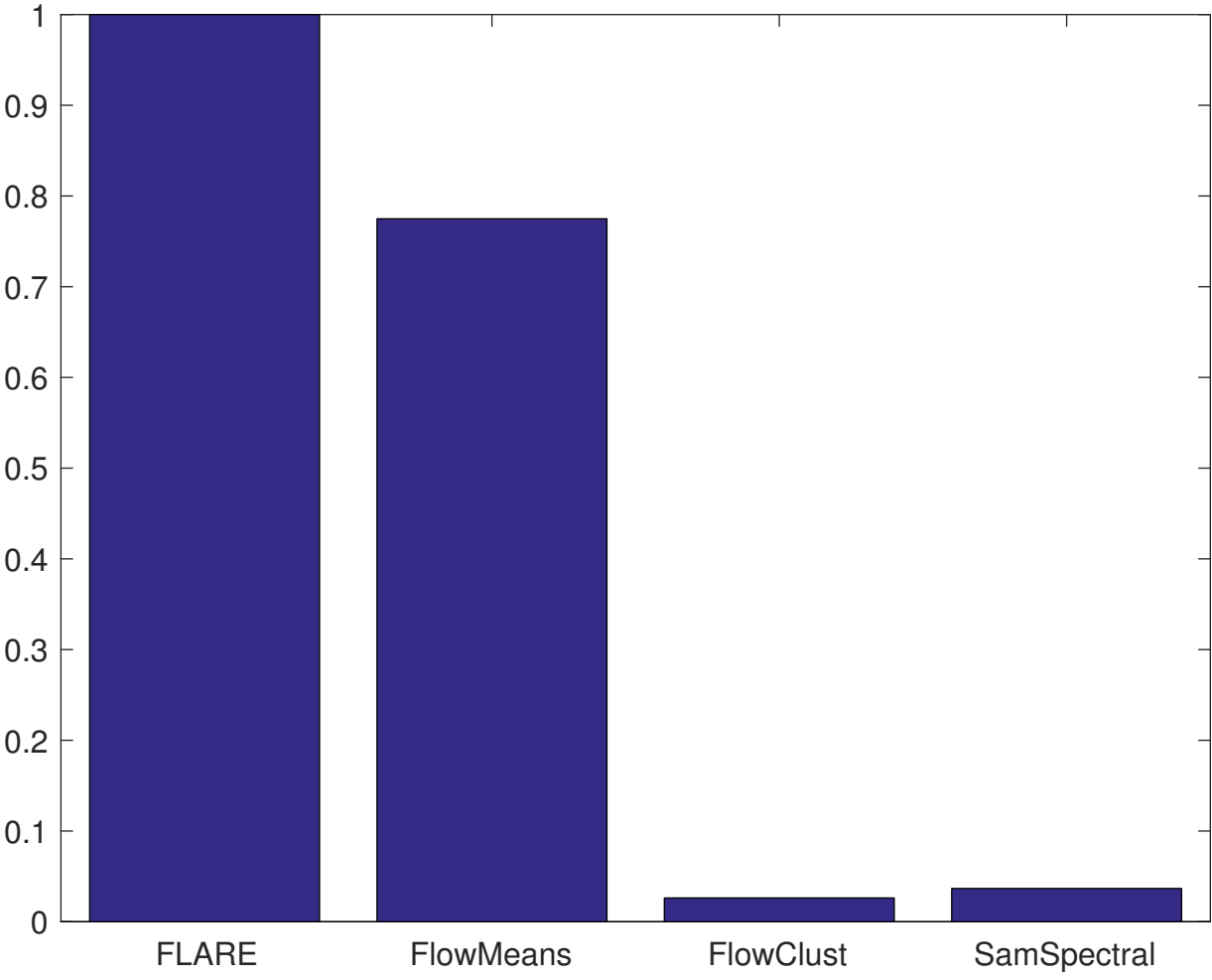

Supplement: S4 Fig — (PDF) [file pone.0228651.s004.pdf]

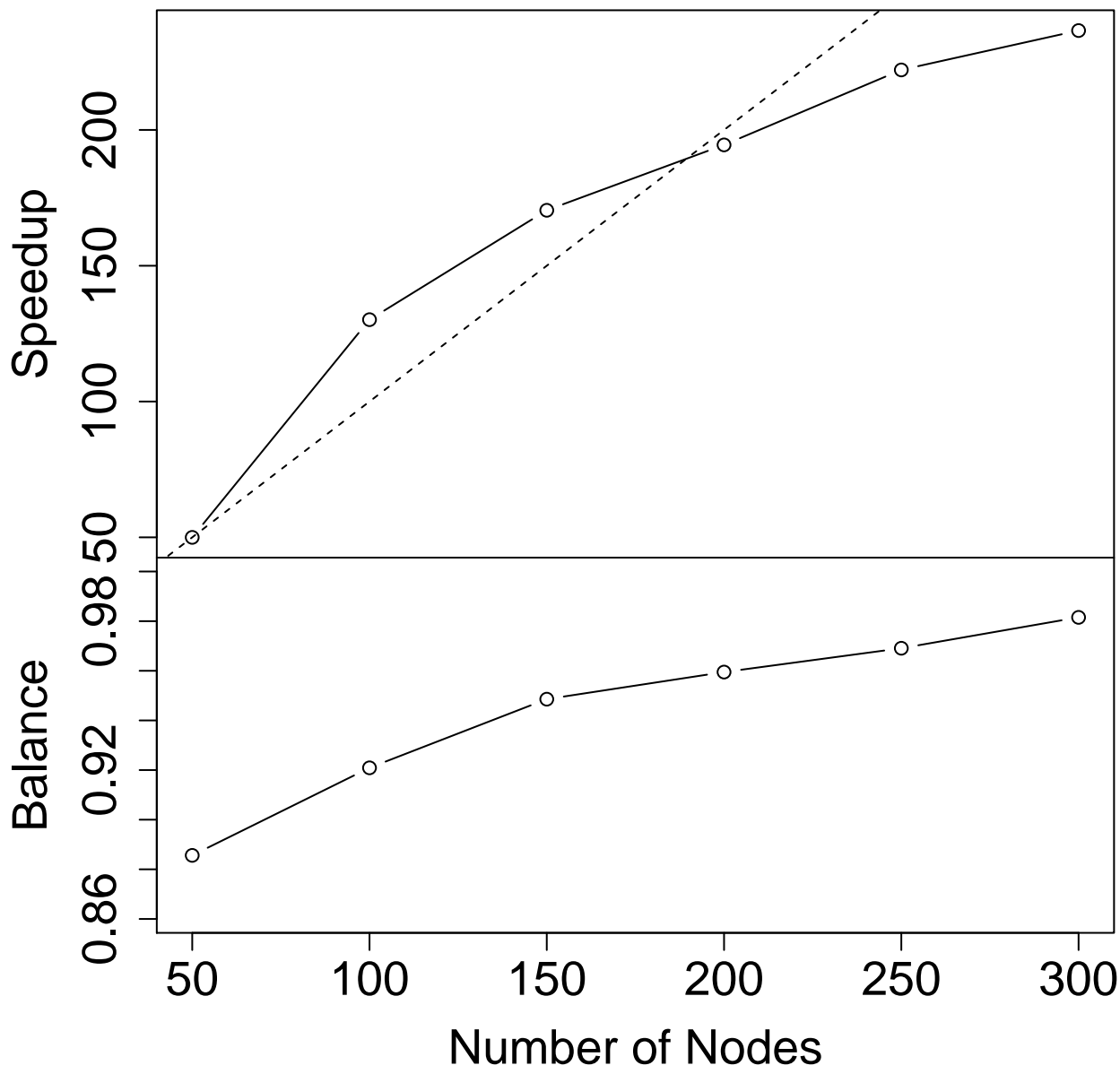

Supplement: S5 Fig — The top panel shows the speedup rate of our parallel inference algorithm using increasingly more cluster nodes. The bottom panel shows the load balancing efficiency. The balancing efficiency is calculated using Eq (32). With more nodes, the data are more evenly distributed so that the balancing efficiency keeps increasing. (PDF) [file pone.0228651.s005.pdf]
