# Supplementary material for: High-speed automatic characterization of rare events in flow cytometric data
Source: PLoS One. 2020 Feb 11;15(2):e0228651. doi: 10.1371/journal.pone.0228651 (PMC7012421; doi:10.1371/journal.pone.0228651)
Supplement: S1 File — Further details on the Experimental Results. (PDF) [file pone.0228651.s006.pdf]

Supplemental Materials for 'High-Speed Automatic Characterization of Rare Events in Flow Cytometric Data' (Y. Qi, et al.)

Contents

A Experimental Results **1**
A.1 Synthetic data 1
A.2 Treg data 2
A.3 Speedup Results 2

A Experimental Results

A.1 Synthetic data

We perform the experiment on synthetic and real flow cytometry datasets.

First, using synthetic datasets where we know the ground truth, we compare our method with three state-of-the-art flow cytometry analysis methods, FlowMeans [1], FlowClust [2] and SamSpectral [3]. FlowMeans is based on the classic Kmeans algorithm; FlowClust uses a multi-variate t mixture model; and SamSpectral uses spectral clustering as its core engine. We set the initial number of clusters K to be the same for all methods. The three comparison algorithms use merging techniques to combine similar clusters after the clustering procedures. With a Dirichlet prior that encourages sparse mixture components, our model automatically prunes unnecessary clusters based on the data. It is worth noting that all the comparison methods do not explicitly differentiate multiple samples to find rare cell clusters. By contrast, our model can identify the common clusters across samples and differentiate rare clusters that may only present in few samples.

In our simulation, we generate three large clusters in three samples. One of the three samples has an additional rare cluster close to one of the large clusters. Each large cluster in one sample has 100 data points, and the rare cluster has only 10 points. The center and variance of the big clusters are generated according to the graphical model in Fig 1 of the main document—we generate the prototype means and covariances by sampling from equations 6 and 7 and use them to sample the means and covariances of each sample by equations 4 and 5. We vary the dimension of the data from 2 to 16 and repeat the experiment 50 times. To assess the accuracy of the clustering result, we compute the Adjusted Rand Index (ARI) [4] between the clustering results and the true clusters. The higher the ARI, the more accurate the clustering results. The average ARIs for each method are reported in S1 Fig. Our method achieved the highest ARI compared to other methods.

We visualize the clustering results of our method and the competing methods with a 2D synthetic dataset in S2 Fig. In this figure, each row is a different clustering method, and each column is a sample. The sample having the rare cluster is in the last column. Different clusters are represented by different colors. Our method is the only method that differentiates the rare cluster from the three large clusters as shown in the fourth column.

A.2 Treg data

We also test our method on a small real-world data set, "Treg" [5]. There are two samples in this dataset. Each sample has $\sim 28,000$ cells. A small artificial T-cell cluster ($\sim$ 300 cells) is added to one of the samples. The task is to identify this small T-cell cluster, i.e. the target cluster. To assess the clustering methods, we compute the Jaccard index between each computed cluster and the target cluster. For a cluster C and the target cluste T, Jaccard index is defined as

$$J(C,T) = \frac{|C \cap T|}{|C \cup T|}. \tag{S1}$$

For each method, we report the maximum Jaccard index computed between the target cluster and the all computed clusters. The result is shown in S3 Fig, with standard error over 10 runs with different initializations. Again, our method outperforms the alternative methods significantly.

The denominator of Jaccard index includes the number of data points in the computed clusters. We are also interested in the proportion of the data points in the target cluster that fall in the computed cluster. To this end, we define *detection accuracy* as the following quantity

$$D(C,T) = \frac{|C \cap T|}{|T|}. \tag{S2}$$

and report the maximum $D(C,T)$ for all the computed clusters C for each method in S4 Fig. FLARE consistently outperforms the other three methods.

A.3 Speedup Results

A big advantage of FLARE is that we are able to analyze millions of cells with our parallel inference algorithm on computer clusters within minutes. We tested this parallel inference algorithm using a cluster called Carter, which is maintained by Purdue's Rosen Center for Advanced computing. Carter consists of a total of 660 nodes, Each of which contains two 8-core Intel Xeon-E5 processors. The nodes each have 56 Gbps FDR Infiniband connections. To facilitate communication between nodes we use the Message Passing Interface (MPI) version 1.4.4. As shown in S5 Fig, we are able to achieve nearly linear speedup: just under 5 seconds for the oncogenic signature data set, 265 seconds for the scaled hematopoiesis data.

References

[1] Aghaeepour, N., Nikolic, R., Hoos, H. H., and Brinkman, R. R. Rapid cell population identification in flow cytometry data. *Cytometry A* **79**(1), 6–13 (2011).

[2] Lo, K., Hahne, F., Brinkman, R. R., and Gottardo, R. flowClust: a Bioconductor package for automated gating of flow cytometry data. *BMC Bioinformatics* **10**(1), May (2009).

[3] Zare, H. and Shooshtari, P. *SamSPECTRAL: Identifies cell population in flow cytometry data*, (2009). R package version 1.4.1.

[4] Rand, W. M. Objective Criteria for the Evaluation of Clustering Methods. *Journal of the American Statistical Association* **66**(336), 846–850 (1971).

[5] Pyne, S., Hu, X., Wang, K., Rossin, E., Lin, T.-I., Maier, L. M., Baecher-Allan, C., McLachlan, G. J., Tamayo, P., Hafler, D. A., and et al. Automated high-dimensional flow cytometric data analysis. *Proceedings of the National Academy of Sciences* **106**(21), 8519–8524, May (2009).
